# Variation in Manifest Subjective Refraction in a Population Screened for Refractive Surgery

**DOI:** 10.3390/diagnostics16091396

**Published:** 2026-05-05

**Authors:** Achim Langenbucher, Jascha Wendelstein, Nóra Szentmáry, Alan Cayless, Anika Förster, Peter Hoffmann, Suphi Taneri

**Affiliations:** 1 Department of Experimental Ophthalmology, Saarland University, 66424 Homburg, Germany; wendelsteinjascha@gmail.com (J.W.); nszentmary@gmail.com (N.S.); 2 Department of Ophthalmology, Ludwig Maximilian University (LMU), 81377 Munich, Germany; 3 Department of Ophthalmology, Semmelweis-University, 1085 Budapest, Hungary; 4 School of Physical Sciences, The Open University, Milton Keynes MK7 6AA, UK; alan.cayless@gmail.com; 5 Zentrum für Refraktive Chirurgie, Augenzentrum am Franziskus-Hospital, 48145 Münster, Germany; foerster@refraktives-zentrum.de (A.F.); taneri@refraktives-zentrum.de (S.T.); 6 Eye-Clinic, Ruhr-University, 44892 Bochum, Germany; 7 Augen- und Laserklinik Castrop-Rauxel, 44575 Castrop-Rauxel, Germany; pupillenpeter@gmx.de

**Keywords:** subjective manifest refraction, power vector analysis, variation in refraction, repeat measurements

## Abstract

**Background/Objectives**: The purpose of this study was to investigate the variation in subjective manifest refraction measures in a patient cohort screened for myopic refractive surgery. **Methods**: In this retrospective non-randomised cross-sectional single-centre study, we evaluated a dataset containing sequences of three refraction measurements performed by four experienced optometrists in 175 eyes screened for refractive corneal or lens surgery for myopia or myopic astigmatism. Refraction was converted from sphere (SPH), cylinder (CYL) and axis to power vector components (spherical equivalent SEQ and cylinder projections C0 and C45). The mean power vectors of the three repeat measurements (MEAN) and the deviations (DEV) of the repeat measurements from the MEAN were evaluated. **Results**: MEAN values for SPH/CYL/SEQ/C0/C45 were −5.93/0.99/−5.44/−0.47/0.02 D and the corresponding standard deviations were 0.20/0.16/0.17/0.17/0.16 D. DEV of both SEQ and CYL correlated significantly with patient age (Spearman R = 0.16 and 0.20). DEV of CYL correlated with mean (myopic) SEQ (R = −0.22) and CYL (R = 0.27) whereas DEV of SEQ showed no significant correlation with mean SEQ or CYL. **Conclusions**: The variation in subjective manifest refraction with repeat measurements is in a range of ±0.16 to ±0.20 D for SPH, CYL and the power vector components SEQ, C0 and C45. If reliable subjective refraction measurements are mandatory, e.g., for planning refractive surgery procedures or for formula constant optimisation, repeat refractometry measures could help to ensure representative data and to estimate the intraindividual variations.

## 1. Introduction

A study on the variation in subjective refraction defines the fundamental measurement uncertainty of refractive surgery outcomes and therefore quantifies the true limits of predictability, comparison, and optimisation in modern refractive care. Every refractive procedure—whether laser or lens-based—ultimately aims to optimise subjective refraction rather than other quantities such as biometry measurements, wavefront RMS, ray tracing, or Zernike polynomials. However, the absolute value of the subjective refraction is often taken as the fundamental truth, with repeatability, variability, or bias rarely quantified. Understanding this intrinsic measurement variability is essential in order to correctly interpret refractive outcomes, define clinically meaningful differences, and estimate the lower bound of achievable prediction accuracy.

Manifest refraction is one of the main measurable outcomes in ophthalmology. It involves both spherical and cylindrical corrections of the eye in order to achieve the best visual function in terms of corrected visual acuity. Subjective refraction is normally measured and reported monocularly, but binocular balancing is ultimately performed to determine the binocular visual acuity. Refraction is measured either objectively using an autorefractometer or subjectively in terms of manifest refraction with a phoropter or using trial glasses in a trial frame. In a clinical context, a distinction is made between distance correction (with a refraction lane distance of 4 to 6 m) and intermediate (workplace) or near (reading)-distance correction. Accurate refractometry is required not only for evaluating the visual function but also for planning refractive surgery procedures [1] or for evaluating the performance of refractive and cataract surgery procedures [2,3].

However, we know that refractometry can exhibit some variation over time resulting from changes in the tear film or fluctuations in biometric parameters or accommodation [4,5,6,7]. These fluctuations may cause diurnal refraction shifts as well as longer-term variations over days, weeks or months. It is also known that environmental conditions, the test strategy, and the optotypes used (e.g., letters, numbers, Landolt rings or Snellen E) can affect the refraction. These variations and uncertainties in refraction measurement act as ‘background noise’ in planning refractive surgery or in evaluating lens power calculation formulae [1]. This is especially relevant in planning refractive procedures such as laser vision correction or implanting phakic or AddOn lenses, where the manifest subjective refraction is directly used for calculating the ablation profile or the power of the lens implant.

Refraction is clinically documented in terms of the sphere, cylinder and axis of the cylinder, where the cylindric correction may be recorded in either plus or minus cylinder nomenclature [8,9]. In the minus cylinder notation, the sphere and axis refer to the power and meridional axis of the principal meridian with the maximum refraction (highest positive or lowest negative), and in the plus cylinder notation, the sphere and axis refer to the power and meridional axis of the principal meridian with the minimum refraction (lowest positive or highest negative) [10].

Refraction is generally processed as a 3D vector, expressed either in classical notation (sphere, cylinder and axis) or as a power vector (spherical equivalent and projection of the cylinder to the 0- and 90-degree and 45- and 135-degree meridians). Given the dependencies of sphere, cylinder and axis, and the 180-degree periodicity of the cylinder axis, data processing and statistics require handling of orthogonal and independent power vector components instead of sphere, cylinder and axis [4,10,11]. This enables the three power vector components to be statistically processed directly and to be visualised in a uniform dioptric space. To facilitate handling of the 3D power vectors, the spherical equivalent is normally considered as a univariate parameter whereas the astigmatic power vector components are evaluated in terms of bivariate statistics [4,8,12,13,14,15]. Clinicians typically use double-angle plots (with an axis range of 0 to 180 degrees) for visualisation since these best represent the 180° periodicity of the astigmatism. In such plots, the X and Y axes refer to the projection of the refractive cylinder to the 0/90-degree and 45/135-degree meridians. In contrast to positional X/Y coordinates (e.g., pupil centre location), the symmetry between left and right eyes may be accounted for by flipping the sign of the Y component of the bivariate cylinder (i.e., by mirroring left eyes) [16,17].

The purpose of the present study is

- To evaluate variations in manifest subjective manual refraction measurements performed with physiological pupil size using an automated phoropter with a sequence of three repeat measurements performed by four experienced optometrists;
- To identify potential influencing factors for the variations in refractometry.

We will achieve this based on a clean dataset containing examinations of untreated eyes screened for laser vision correction or refractive lens surgery with a corrected distance visual acuity of at least 0.63 decimal (0.2 logMAR).

## 2. Materials and Methods

1. Dataset for our evaluation and manifest refraction protocol

A dataset containing 3 independent repeat measurements for each of 175 eyes (in total *N* = 525 measurements) taken prior to laser vision correction or refractive lens surgery was considered in this study. Soft contact lens wear was discontinued for at least 2 weeks and rigid contact lens wear for at least 4 weeks before the initial examination. In cases where contact lenses were used daily or overnight, the contact lens wear cessation period was doubled. The study group consisted of myopic patients scheduled for refractive surgery and otherwise healthy with no indications of tear film pathologies, corneal irregularities or other contraindications for refractive surgery. All subjective manifest refraction measurements were performed between 1 April 2015 and 1 February 2019 at the Augenzentrum am St. Franziskus-Hospital (Münster, Germany) using a Visutron phoropter (Möller-Wedel Optical GmbH, Wedel, Germany) with a refraction lane distance of 6 m under standardised measurement conditions (identical lighting and optotype screen) in the same examination room. The 3 repeat refraction measurements for each eye including recording of the corrected visual acuity (in decimal values) were performed on 3 different days by 3 different optometrists, each with more than 4 years of experience, using Landolt ring optotypes. The pupils were not affected by pharmacological dilation or by cycloplegia. No binocular balancing was employed as the aim was to establish the optimum refraction for each eye prior to refractive surgery. The cylinder correction (magnitude and axis) was fine-tuned by means of a cross-cylinder adjustment using a “sun dial”. The details of the measurement protocol are reported in Taneri et al. 2020 [1]. The data were transferred to us in anonymised form for scientific evaluation. The local ethics committee (IRB) has provided a waiver for this study (Ärztekammer des Saarlandes, 157/21), as all data processed in this study were already anonymised at source before being transferred to us for processing. This precludes any back-tracing of the identity, and therefore informed consent of the patients was not necessary. Data tables were reduced to the relevant parameters required for our data analysis, consisting of the following measurements: patient ID, patient age (in years) at the time of examination, the laterality (left or right eye), gender (male or female), and manifest refractions (1, 2 and 3) in terms of sphere (SPH in dioptres (D)), cylinder (CYL in D, minus cylinder nomenclature) and cylinder axis (A in degrees). None of the patients required any prismatic correction.

2. Data processing in Matlab (Version 2025b)

Refraction (SPH, CYL, A) was transformed from minus cylinder to plus cylinder notation (SPH: = SPH + CYL; CYL: = −CYL; A: = mod(A + 90,180)) and converted to power vectors (with components SEQ, C0, C45, all in D [16,17]) using

$$\begin{array}{ccc} SEQ & = & SPH+0.5\cdot CYL\\ C0 & = & CYL\cdot cos(2\cdot A).\\ C45 & = & CYL\cdot sin(2\cdot A) \end{array}$$

In this context, SEQ refers to the mean refractive correction which corresponds to the location of the circle of least confusion in the object space, and C0 and C45 refer to the cylindrical power vector components projected to the 0- and 90-degree meridians and to the 45- and 135-degree meridians, respectively. The mean refraction power vector (with vector components SEQmean, C0mean, C45mean, all in D) was derived by averaging the corresponding power vector components of the 3 refraction measurements per eye, and then re-converting to the standard notation of sphere, cylinder and axis (SPHmean, CYLmean and Amean, all in D) [15,16,17]. In the next step, we derived the difference of each of the 3 repeat measurements from their mean (power vector components SEQdev, C0dev and C45dev, all in D) by subtracting the individual power vector components (SEQmean, C0mean and C45mean) from the corresponding mean power vector components (SEQ, C0, C45) for each of the 3 repeat measurements [2,12]. In the last step, the mean power vectors (SEQmean, C0mean, C45mean) and deviations from the mean (SEQdev, C0dev and C45dev) were re-converted to standard notation (SPHmean, CYLmean, Amean and SPHdev, CYLdev, Adev) using

$$\begin{array}{ccc} {CYL}_{\left(.\right)} & = & \sqrt{{C0}_{\left(.\right)}^{2}+{C45}_{\left(.\right)}^{2}}\\ {SPH}_{\left(.\right)} & = & {SEQ}_{\left(.\right)}-0.5\cdot {CYL}_{\left(.\right)}\cdot cos(2\cdot A_{\left(.\right)})\\ A_{\left(.\right)} & = & atan(\frac{{C45}_{\left(.\right)}}{{C0}_{\left(.\right)}}) \end{array},$$

where (.) refers to mean or to dev, respectively. Since the axis orientation A_(_._)_ for very small values of CYL_(_._)_ is undefined, we did not include axis values for CYL_(_._)_ < 0.1 D [16].

From the mean (SEQmean, C0mean and C45mean) and deviation from mean (SEQdev, C0dev and C45dev) power vectors, we derived the defocus equivalent (DEQ_(_._)_ in D) as a metric for image blur [4,12] using

$${DEQ}_{\left(.\right)}=\sqrt{{SEQ}_{\left(.\right)}^{2}+{0.25\cdot C0}_{\left(.\right)}^{2}+{0.25\cdot C45}_{\left(.\right)}^{2}}$$

where (.) refers to either ‘mean’ or ‘dev’.

Refractive cylinder axes A were categorised into with-the-rule (wtr, axis of the plus cylinder within 60 and 120 degrees), against-the-rule (atr, axis of the plus cylinder within 0 and 30 or within 150 and 180 degrees), and oblique (obl, cylinder axis within 30 and 60 or within 120 and 150 degrees). The corrected distance visual acuity (CDVA) was converted from decimal values to logMAR units for statistical evaluation.

3. Statistical evaluation

The within-subject standard deviation Sw was taken as a measure for the consistency of the 3 repeat measurements [12] and was derived from

$$Sw=\sqrt{\frac{1}{3}\cdot \left(\left(.\right)dev1^{2}+\left(.\right)dev2^{2}+\left(.\right)dev3^{2}\right)}$$

where (.)dev1, (.)dev2 and (.)dev3 refer to the deviations of the 1st, 2nd and 3rd measurement from their mean, respectively, and (.) refers to SPH, CYL, SEQ, C0, C45, DEQ, and logMAR CDVA. The corresponding precision was documented as 1.96∙Sw, based on a 95% confidence interval.

For statistical analysis, the power vectors were separated into a univariate evaluation of SEQ and a bivariate evaluation of the C0 and C45 components [15,16]. Univariate normality was tested using the Shapiro–Wilk test and bivariate normality by means of the Henze–Zirkler test. Depending on data normality, we used barplots for univariate parameters (SEQ) with arithmetic mean and error bars showing the standard deviations (SD), or violin plots showing the median, quartiles (25% and 75% quantiles), and the 95% confidence intervals, together with the arithmetic means. The bivariate parameters (refractive cylinder with C0 and C45) are visualised using double-angle plots showing the centroids and 95% error ellipses extracted from the variance-covariance matrix or medoids and 95% confidence regions, depending on bivariate normality. Medoids and confidence regions were calculated using iterative convex hull stripping techniques [16].

Interactions between the deviation from mean power vectors (SEQdev, C0dev and C45dev) and patient age, SEQmean, CYLmean and DEQmean, respectively, were performed using the Spearman rank correlation coefficient, with a significance level of *p* < 0.05 being considered statistically significant.

## 3. Results

A total of *N* = 175 eyes from 91 patients with three repeat measurements each were enrolled in this study (112 eyes from female and 63 eyes from male patients; 86 right and 89 left eyes). The mean age was 42.25 ± 10.12 years (median 41.20 years, 95% confidence interval from 27.65 to 61.99 years). Table 1 shows the descriptive data of the mean values for the three repeat measurements and the deviations of the three repeat measurements from the mean value. The SD of CDVA with the three repeat measurements was 0.045 (mean Sw 0.034).

**Figure 1:**
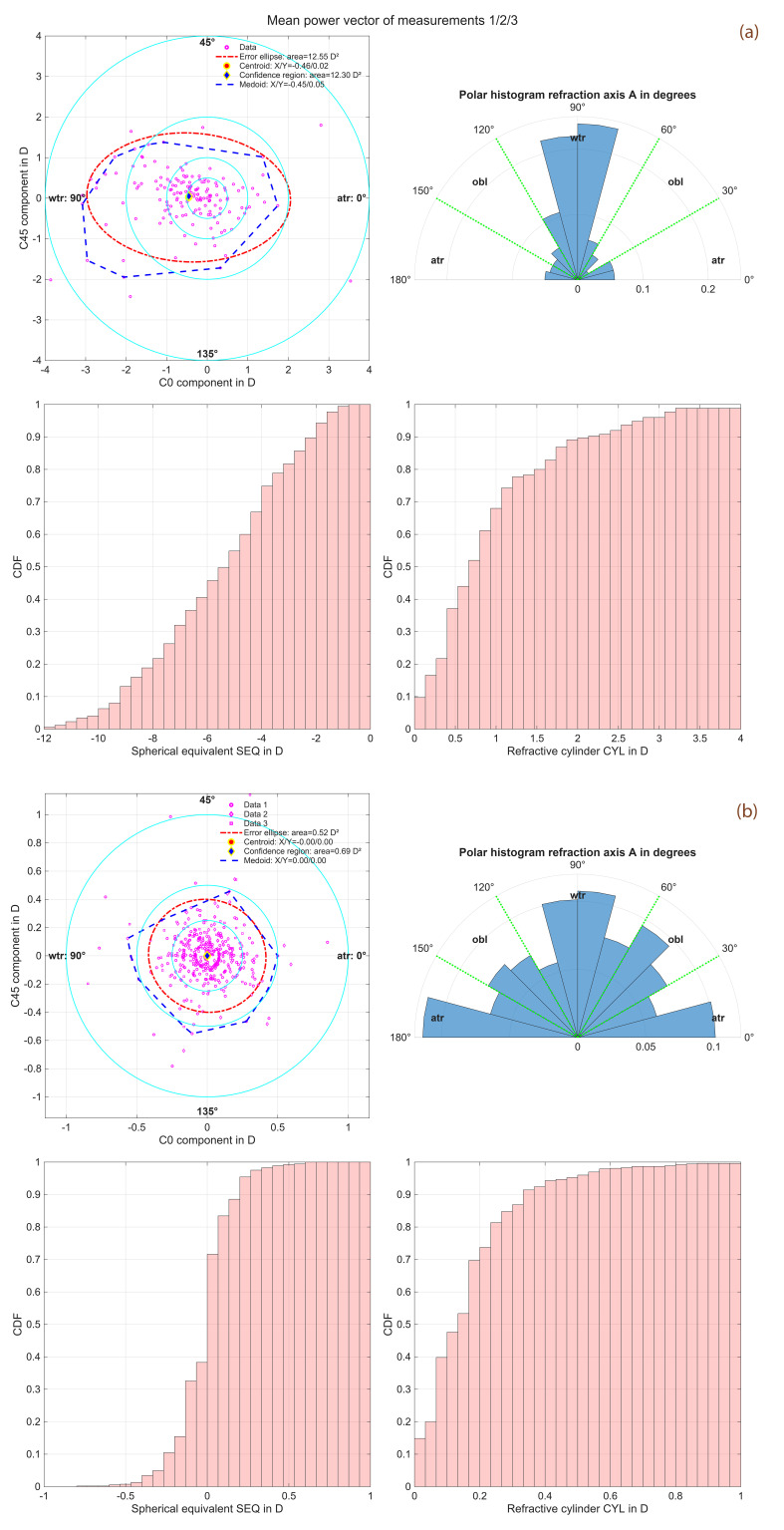
Distributions of the mean values from the 3 repeat measurements for the corneal power vector components (*N* = 175 eyes, (**a**), and the corresponding distributions of the deviations of the 3 repeat measurements from the mean values for the corneal power vector components (*N* = 525 measurements, (**b**). The upper left graphs display the double-angle plot showing the projections of the refractive cylinder CYL to the 0/90-degree (X axis) and the 45/135-degree (Y axis) component, together with the parametric evaluation, with the centroid and the 95% confidence ellipse derived from the variance-covariance matrix and the nonparametric evaluation with the medoid and the 95% confidence region extracted using iterative convex hull stripping. The upper right graphs show the angular distribution of the plus cylinder axis. Please note that the axis for refractive cylinder values <0.1 D has been omitted. The lower left graphs show the cumulative distribution function (CDF) for the spherical equivalent refraction (SEQ), and the lower right graphs show the CDF for the refractive cylinder (CYL). The categorisation of data into with-the-rule (wtr, plus cylinder notation, axis between 60 and 120 degrees), against-the-rule (atr, axis between 0 and 30 or between 150 and 180 degrees), and oblique axes (obl, axis between 30 and 60 or between 120 and 150 degrees) is overlaid on the upper right graphs.

Figure 1a,b provide graphical representations of the distributions of the mean power vectors (1a) and of the vector deviations of the individual measurements from the mean (1b), allowing visualisation in terms of both power vectors and standard notation. In each case, the polar plot (top left) shows the power vector distribution plotted in terms of the C0 and C45 components, with the three other plots depicting the corresponding standard notation parameters A (Axis), SEQ and CYL.

Specifically, Figure 1a displays the distributions of the mean values of the three repeat measurements for the refraction data. The upper left graph shows the double-angle plot with the projections of CYL (C0 on the X axis and C45 on the Y axis) together with the centroid and the 95% confidence ellipse and the medoid and the 95% confidence region. The upper right graph shows the angular distribution of A for CYL ≥ 0.1 D. The lower left and right graphs show the CDF for the SEQ and CYL together with a categorisation of the datapoints into wtr, atr and obl refractive cylinder axes. The centroid and the medoid for the cylindric power vector components in the upper left graph show a mild cylinder with-the-rule (X/Y coordinates: 0.46/0.02 D and 0.45/0.05 D). The larger scatter in X (indicated by the 95% confidence ellipse and the confidence region) indicates a larger variation in the cylinder data with-the-rule and against-the-rule as compared to the oblique axes. As shown in the polar histogram in the upper right graph, the majority of the refractive cylinder axes are in a range around 90 degrees indicating a cylinder with-the-rule. From the CDF plot in the lower right graph, we see that 68%/89% of eyes show a mean refractive cylinder of up to 1.0/2.0 D.

Figure 1b displays the corresponding distributions of the deviations of the three repeat refraction measurements from their mean values. Both the centroid and the medoid for the cylindric power vector components in the upper left graph indicate a tight and mainly symmetric distribution around the origin (X/Y coordinates: 0.00/0.00 D for centroid and medoid). The larger area of the 95% confidence region as compared to the confidence ellipse (0.69 vs. 0.52 D^2^) indicates some extreme values in the C0 and C45 distributions. As shown in the polar histogram in the upper right graph, the axis distribution of the deviation from the mean is more homogeneous compared to the axis distribution of the mean axis for the three repeat measurements. From the CDF plot in the lower right graph, we see that 70% of the CYL repeat measurements are within 0.2 D of variation.

**Figure 2:**
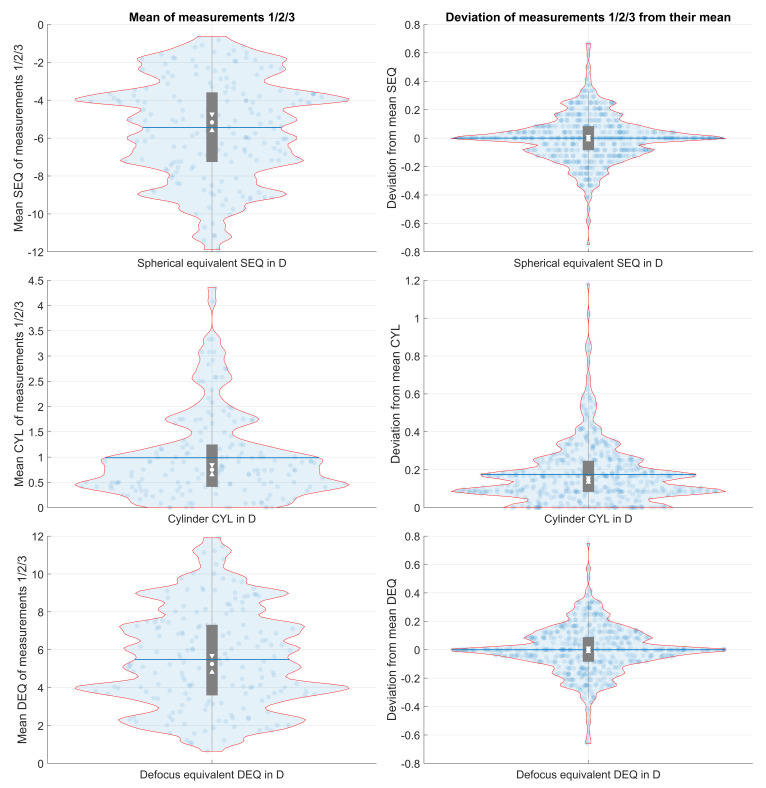
Distributions of mean values of the 3 repeat measurements (graphs on the left, 175 datapoints) and the corresponding distributions of the deviations of the 3 repeat measurements from the mean value (graphs on the right, 525 datapoints) for the spherical equivalent refraction (SEQ, first row), the refractive cylinder (CYL, plus notation, second row), and the defocus equivalent (DEQ, last row). In addition to the Kernel distributions (Kernel size 0.1 D for the left and 0.01 D for the right graphs), the violin plots also show the arithmetic mean (blue lines), the quartiles (black bar), the median (white dot) and the 95% confidence intervals for the median (notches).

Figure 2 uses violin plots to visualise the distributions of the standard notation parameters SEQ and CYL and of the defocus equivalent DEQ. Specifically, the violin plots in the left column of Figure 2 show the kernel distributions of the mean values of the three repeat measurements and the plots in the right column show the corresponding distributions for the deviations of the three repeat measurements from the mean value, in each case, for the SEQ (first row), CYL (second row), and DEQ (last row). The arithmetic mean (blue lines), the quartiles (black bar), the median (white dot) and the 95% confidence intervals for the median (notches) are overlaid on the violin plots. Comparing the upper left and the lower left violin plots indicates a large similarity between SEQ and DEQ (with opposite sign). This means that the image blur characterised by the DEQ is determined mostly by the SEQ whereas the CYL plays only a minor role. The middle left violin plot indicates a large concentration of low and moderate CYL values up to 1.5 D but with some tails up to 4.5 D.

**Figure 3:**
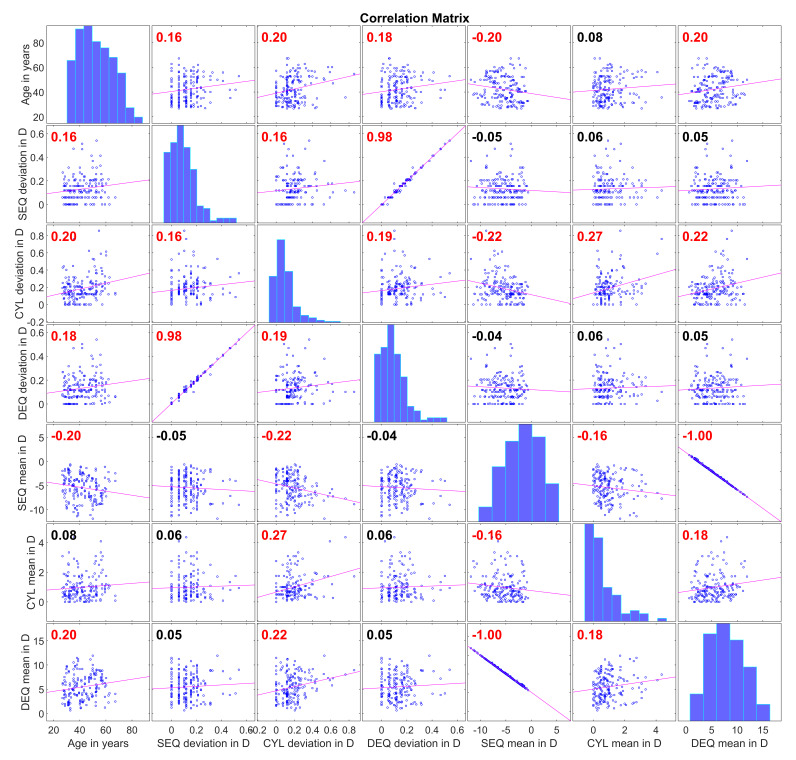
Correlation plots for the patient age, the deviations of the 3 repeat measurements from their mean (dev) and the mean of the 3 repeat measurements (mean) for the spherical equivalent refraction (SEQ), the refractive cylinder (CYL, plus notation), and the defocus equivalent (DEQ) as a measure of the image blur. On the diagonal, the histograms show the distributions for the parameters, and on the off-diagonal, the scatterplots show the pairwise correlations together with the linear regression line (red line) and the Spearman rank correlation coefficient (shown in each plot). Correlation coefficients in red indicate a statistically significant correlation (significance level < 0.05).

Figure 3 aims to visualise the correlations between parameters. Specifically, the correlation plots in the grid represent the pairwise relationships between the patient age, the mean values of the three repeat measurements for SEQ, CYL and DEQ, and the deviations of the three repeat SEQ, CYL and DEQ measurements from their means. The graphs on the diagonal of the matrix plot show the histogram distributions for the individual parameters, and the graphs on the off-diagonal show the pairwise correlations together with the linear regression line (red line) and the Spearman rank correlation coefficient (values in red indicate statistically significant correlations). From the first column of correlation plots, we can see that the variation in the three repeat measurements increases with age for SEQ, CYL and DEQ. From the 2nd to 4th row and the last three columns of the correlation plots, we understand that the variation in SEQ and in DEQ is not related to the mean SEQ, CYL and DEQ, whereas the variation in CYL decreases with the mean SEQ and increases with mean DEQ (both refer to an increase in myopia). The variation in CYL increases especially with the mean CYL. This means that the variability of CYL measurement becomes worse with higher refractive cylinder values.

## 4. Discussion

Subjective manifest refraction and corrected visual acuity are the most frequently reported outcome measures in ophthalmology. DIN:ISO8596:2020 describes the standards for determining visual acuity with and without refractive correction [18]. Reliable refraction for distant objects is crucial for planning all refractive surgery procedures [1] but also for evaluation of the outcome of refractive surgery procedures [4,12,16]. While manifest subjective refraction is one of the main input parameters in refractive surgery planning, it should always be interpreted in conjunction with cycloplegic refraction and a comprehensive preoperative evaluation, particularly in younger patients or in eyes with suspected accommodative influences, in order to ensure an accurate and clinically robust refractive target. It is also known from clinical practice that refractometry shows a degree of both short-term and long-term variability [19]. Since obtaining subjective refractometry by an experienced ophthalmologist, optometrist or optician is quite time-consuming, most of the clinical data on variation in repeat refraction measurements are derived from objective refractometry (autorefractors or wavefront sensors [1,8,9,11,20,21,22,23,24,25,26]), and the repeatability of subjective manifest refraction [1,8,27,28,29], especially as performed by different examiners (inter-observer variability), is rarely reported.

The aim of the present study was to evaluate the consistency of subjective manifest refraction measurements performed by experienced optometrists in a highly selected study population screened for refractive corneal or lens surgery for myopia or myopic astigmatism with minimum 0.2 logMAR corrected visual acuity. All study participants were measured according to the same strict protocol on three different days by one or more out of four experienced optometrists [1].

Since only eyes screened for myopic refractive correction were included, the SEQ of all eyes was negative with a range between −12.0 and −1.0 D [1]. In contrast to the previous study of Taneri et al., who considered all eyes with two or more repeat measurements, we considered eyes with a sequence of precisely three repeat measurements. This allowed Sw to be extracted and used as a relevant metric for variability, e.g., for error propagation models.

The main findings are that variations in the order of 0.16–0.17 D in SEQ, CYL and DEQ are possible with repeat measurements, with the defocus equivalent DEQ correlated primarily with the spherical equivalent SEQ. These variations appear larger in eyes with higher ametropia and in older patients. This suggests that, while time-consuming, there could be some benefit to repeat measurements of subjective manifest refraction, especially in elderly patients and those with higher prescriptions.

From the double-angle plot and the polar histogram in Figure 1a, we see that the axis of the refractive cylinder in most eyes (in plus notation) ranged around 90 degrees (indicating a with-the-rule astigmatism). Even though the cylinder did not show bivariate normality, the areas of the 95% confidence ellipse and the 95% confidence region were quite similar (12.55 D^2^ vs. 12.30 D^2^). From the CDF plot, we understand that most of the CYL values (96%) ranged between 0.0 D and 3.0 D.

However, the variations in the three repeat measurements from their mean values are even more interesting than the mean refraction values themselves. We found that even though a refractive cylinder with-the-rule dominates the axis distribution (i.e., the mean C0 component of the cylinder larger than the C45 component), the variation between the three repeat measurements seems to be quite similar for both vector components. However, the 95% confidence region exceeds the 95% confidence ellipse size indicating a tailed distribution of the variation in the refractive cylinder. Further, in this clean dataset, the mean variation in CYL is 0.17 ± 0.16 D and the variations in C0 and C45 (0.0 ± 0.17 D and 0.0 ± 0.16 D) are similar. The variation in SEQ is in the same range (0.00 ± 0.17 D) as the variations in C0, C45 and the variation in DEQ (0.00 ± 0.17 D) which characterises the blur of the uncorrected retinal image.

The distribution of mean DEQ appears to be a vertically flipped version of the distribution of mean SEQ, suggesting that the blur of the uncorrected image is determined mostly by the SEQ whereas the CYL plays a minor role. Accordingly, from the kernel distributions of the variations in SEQ and DEQ, we can see that the DEQ distribution is similar to the vertically flipped SEQ distribution.

From Figure 3, we learn that in our study population, the variations in SEQ, CYL and DEQ measurement increase systematically with patient age. This might be of interest as it would be reasonable to assume that in a younger population, which typically shows a larger physiological accommodation, the variation could be larger as compared to an elderly population with degraded accommodation. Future studies should therefore be performed to evaluate whether this effect also holds in an emmetropic or hyperopic study population. However, this suggests that repeat measurements could be beneficial to derive more reliable refraction values in situations such as spectacle prescription, especially in elderly patients. Even more interestingly, the variations in SEQ and DEQ measures do not depend on the mean SEQ, mean CYL and mean DEQ. In contrast, the data show that the variation in CYL systematically increases with the mean (myopic) SEQ error (which in our study population is equivalent to an increase in mean DEQ) and with the mean CYL. This means that the reliability of refractometry seems to drop with larger ametropia (both SEQ and CYL).

If we compare our results to previous studies focusing on the variations in subjective refraction with repeat measurements, we find, for example, that our data are mostly consistent with the results of Rosenfield & Chiu 1995 who reported standard deviations of ±0.14 D and ±0.18 D for subjective and objective refractive sphere and cylinder values [29]. In the present study, the standard deviations for SEQ and CYL (0.21 and 0.16 D) were slightly larger. Shah et al. performed an interesting study based on three ‘standardized patients’ who were trained to provide accurate responses during refractometry. For the three standard patients, the 95% confidence intervals for measurements performed by different optometrists ranged between 0.29 and 0.53 D for the SEQ, 0.18 to 0.43 D for C0, and 0.22 to 0.47 D for C45. These findings are comparable with our results: multiplying our standard deviations (SEQ (0.17 D), C0 (0.17 D) and C45 (0.16 D)) by 1.96 for a 95% confidence interval gives values of 0.47 D for SEQ and C0, and 0.44 D for C45.

However, our study has some limitations: (1) the monocentric study population includes highly selected patients (with a visual acuity of 0.63 decimal or higher) screened for refractive surgery. This study population may not reflect the general condition of a wider population in an outpatient department. (2) All patient eyes were examined monocularly by 3 out of 4 experienced optometrists (inter-observer variation). In a real-life scenario, we argue that refractometry might quite commonly be performed by the same examiner (intra-observer variation), potentially resulting in smaller variations between the measures due to some ‘memory effect’. (3) In this study, we did not consider other biometric parameters such as keratometry or axial length, and we did not directly measure the vertex distance between corneal vertex and correction glasses. These parameters might also affect the variability of refractometry. However, examiners had access to automated refraction, wavefront refraction and corneal astigmatism as well as prior (spectacle) refraction when measuring the refraction. (4) As the purpose of our study was the description of variability in subjective manifest refraction rather than statistical testing or comparisons, we did not carry out any statistical tests for correlations between eyes or other statistical tests for correlated data. Where both eyes of a patient were listed for a refractive procedure, we treated each eye as a separate case.

Clinical impact: The demonstrated repeatability limits (≈±0.16–0.20 D) indicate that subjective manifest refraction is not a true “point value” but a distribution, and this should be explicitly considered when planning and evaluating refractive procedures. In clinical practice, averaging at least two or three measurements is advisable—particularly in older patients and in eyes with higher spherical or cylindrical errors—in order to improve robustness and avoid overinterpretation of small inter-visit differences (<0.25 D). Incorporating this intrinsic variability into nomogram design and outcome benchmarking may help to define realistic accuracy targets and reduce the risk of systematic overcorrection or unnecessary enhancements.

## 5. Conclusions

In conclusion, this study evaluates the consistency of subjective manifest refractometry measurements in a highly selected patient cohort screened for refractive surgery. We found that, even with a highly standardised study setup, the standard deviations of the sequences of three repeat measurements are of the order of 0.16 to 0.17 D for SEQ, C0, C45 and CYL, and 0.20 for SPH. Since the variation in refractive cylinder seems to increase with the (myopic) spherical equivalent refraction and refractive cylinder, refractometry should be repeated in eyes with larger ametropia. Further multicentric studies with a larger sample size could help to improve our understanding of the factors affecting variations in refractometry. In situations where reliable subjective refraction is mandatory, e.g., for planning of refractive surgery procedures at the cornea or lens or for formula constant optimisation, repeat refractometry measures could help to ensure a more reliable manifest refraction and to understand the intra-eye variability of refraction.

## Figures and Tables

**Table 1:** Explorative data for the mean values of the 3 repeat measurements (upper part) and deviations from the mean values (lower part) of refraction in terms of sphere (SPH), plus cylinder (CYL), spherical equivalent (SEQ), cylindric power vector components (C0 and C45), together with the defocus equivalent (DEQ) and the logMAR corrected visual acuity (logMAR). Mean, SD, median, and 2.5% and 97.5% quantile refer to the arithmetic mean, standard deviation, median, and the lower and upper boundaries of the 95% confidence intervals. The lower part of the table displays the mean and lower and upper boundaries of the 95% confidence intervals (CIs) for Sw and the precision derived from the 3 repeat measurements of each eye.

Triple Measurement in 175 Eyes		Standard Notation: Sphere and Cylinder		Defocus Equivalent	Corrected Visual Acuity	Power Vector Components		
Data in D		SPH	CYL	DEQ	logMAR	SEQ	C0	C45
Mean of measurements		SPHmean	CYLmean	DEQmean	logMARmean	SEQmean	C0mean	C45mean
*N* = 175 datapoints	Mean	−5.931	0.985	5.487	−0.087	−5.439	−0.458	0.019
	SD	2.683	0.843	2.5763	0.109	2.596	1.026	0.650
	Median	−5.7500	0.7500	6.251	−0.100	−5.167	−0.356	0.066
	2.5% quantile	−11.558	0.0	1.219	−0.233	−10.760	−2.885	−1.748
	97.5% quantile	−1.569	3.114	10.829	0.171	−1.198	1.380	1.330
Deviation from mean		SPHdev	CYLdev	DEQdev	logMARdev	SEQdev	C0dev	C45dev
*N* = 525 datapoints	Mean	−0.009	0.175	0.000	0.000	0.000	0.000	0.000
	SD	0.2050	0.1590	0.170	0.045	0.169	0.170	0.164
	Median	−0.002	0.146	0.000	0.000	0.000	0.000	0.000
	2.5% quantile	−0.419	0.000	−0.339	−0.079	−0.333	−0.334	−0.365
	97.5% quantile	0.414	0.584	0.333	0.079	0.333	0.331	0.323
Within-subject standard deviation Sw and precision								
*N* = 175 datapoints	Mean Sw	0.169	0.186	0.133	0.034	0.132	0.126	0.113
	95% CI of Sw	0.00 to 0.436	0.00 to 0.592	0.00 to 0.419	0.00 to 0.094	0.00 to 0.419	0.00 to 0.421	0.00 to 0.403
	Precision	0.332	0.365	0.262	0.067	0.2581	0.248	0.221
	95% CI of precision	0.00 to 0.855	0.00 to 1.161	0.00 to 0.821	0.00 to 0.185	0.00 to 0.822	0.00 to 0.826	0.00 to 0.790

## Data Availability

The data presented in this study are available on request from the corresponding author due to privacy reasons.
